# Supplementary material for: Multifunctional electrolyte additive for high power lithium metal batteries at ultra-low temperatures
Source: Nat Commun. 2025 Apr 8;16:3344. doi: 10.1038/s41467-025-58627-3 (PMC11978743; doi:10.1038/s41467-025-58627-3)
Supplement: Supplementary file 1 — Supplementary Information [file 41467_2025_58627_MOESM1_ESM.pdf]

Supplementary Information

Multifunctional electrolyte additive for high power lithium metal batteries at ultra-low temperatures

Weili Zhang*[1,2,†], Yang Lu[1,†], Qingqing Feng[2,†], Hao Wang[2], Guangyu Cheng[3], Hao Liu[2], Qingbin Cao[4], Zhenjun luo[2], Pan Zhou[1], Yingchun Xia[1], Wenhui Hou[1], Kun Zhao[2], Chunyi Du[2], Kai Liu*[1]

[1]Department of Chemical Engineering, Tsinghua University, China

[2] Tsinghua University Hefei Institute for Public Safety Research, Hefei, China

[3]State Key Laboratory of Space Power-Sources, Shanghai Institute of Space Power-Sources, Shanghai 200245, China

[4]Xinyuan Qingcai Technology Co., Ltd, Beijing, China

[†]These authors contributed equally: Weili Zhang, Yang Lu, Qingqing Feng

*Corresponding author: E-mail: liukai2019@tsinghua.edu.cn; zhangweili@tsinghua-hf.edu.cn

Supplementary Note 1: Exploration of the Reaction Mechanism between Additives and Lithium Metal

To elucidate the reaction mechanism between the synthesized additive and lithium metal, we systematically immersed lithium metal foils in an excess of 0.1 M PN-DME solution for varying durations. Subsequently, we performed XPS analysis to quantitatively assess the evolution of SEI layer thickness on the lithium metal surfaces under different reaction time. As shown in the Fig. S1, when the immersion time was 1 hour, the lithium metal signal was detected at a sputtering depth of 20 nm. When the immersion time was extended to 2 hours, the lithium metal signal was not appeared until the sputtering depth reaches 50 nm. Further prolonging the immersion time did not significantly increase the SEI thickness, as the lithium metal signal was still detectable at a sputtering depth of 50 nm. Based on these observations, we propose that the formation of a stable SEI layer through the reaction between PN and lithium metal requires a certain duration (≥2 hours). Once the SEI layer is pre-formed on the lithium metal surface, it effectively prevents further reactions between the lithium metal and electrolyte components. Therefore, the thickness of the in situ formed SEI layer can be effectively controlled by regulating the resting time after battery assembly.

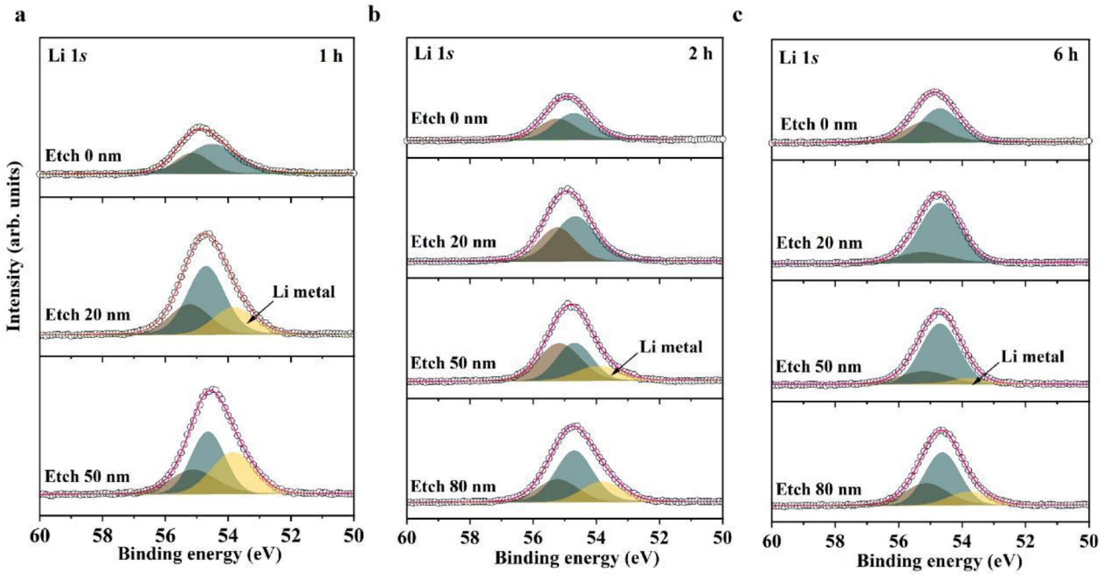

Fig. S1. XPS characterization of SEI layer thickness. Li 1*s* XPS depth profiles of immersed lithium metal in a DME solution containing 0.1 M PN for (a) 1h, (b) 2h, and (c) 6h. Source data are provided as a Source Data file.

Supplementary Note 2: Exploring the stability of additives and electrolytes

To assess the long-term storage stability of the synthesized additive, we re-examined the PN additive synthesized 6 months prior through comprehensive ^{1}H and ^{19}F nuclear magnetic resonance (NMR) spectroscopic analysis. As illustrated in Fig.S2 and Fig.S3, both ^{1}H and ^{19}F resonance peaks maintained identical chemical shift positions and intensity distributions after prolonged ambient-temperature storage, demonstrating no detectable chemical degradation or structural evolution. This remarkable spectral consistency conclusively confirms the exceptional shelf stability of the PN additive under routine storage conditions over extended periods.

Regarding the long-term storage stability of ether-based electrolytes containing PN additives, we compared the ^{19}F NMR spectra of a pre-synthesized electrolyte stored for six months with that of a freshly prepared electrolyte (Fig. S4). The results showed that the fluorine NMR peaks of the electrolyte containing PN additives after six months storage under room temperature remained consistent with those of the freshly prepared electrolyte. This demonstrates the stability of the electrolyte during long-term storage at room temperature. Furthermore, to evaluate the aging resistance of the electrolyte system, we subjected both 6-month-aged and freshly prepared electrolyte formulations to rigorous moisture and acid content analysis via Karl Fischer titration and Bromothymol Blue Indicator titration, respectively. As summarized in Table S1, the aged electrolytes exhibited negligible variation in moisture levels and acid content compared to their freshly prepared counterparts after 6 months of storage under ambient conditions. All parameters remain within the industrial acceptance threshold range, collectively confirming the excellent chemical inertness and hydrolytic stability of this electrolyte system during long-term storage.

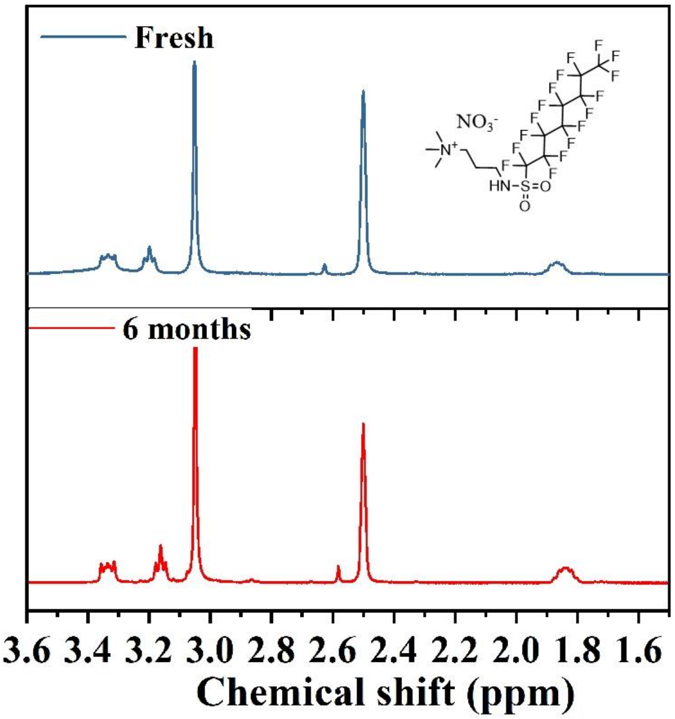

Fig. S2. Comparison of NMR Spectra of PN Additives. ^{1}H NMR spectra of PN stored at room temperature for 6 months and fresh PN. Source data are provided as a Source Data file.

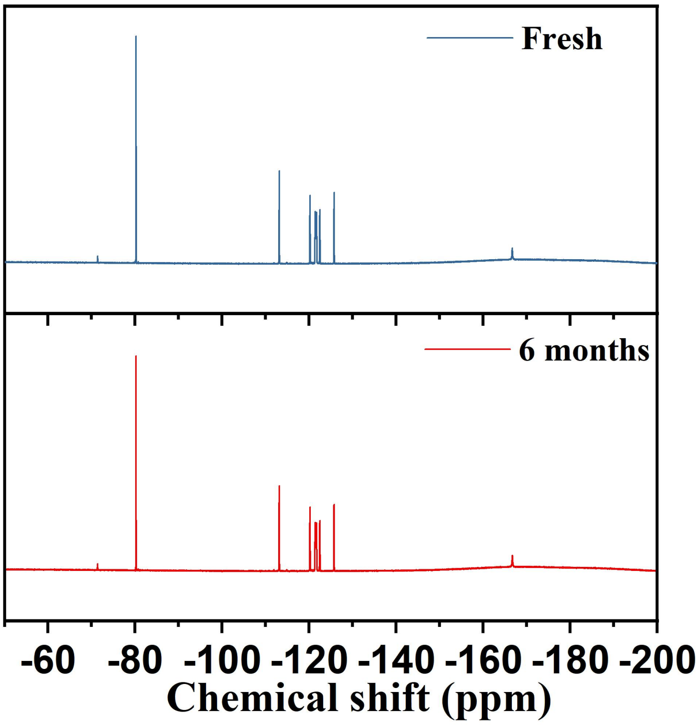

Fig. S3. Comparison of NMR Spectra of PN Additives. ^{19}F NMR spectra of electrolytes containing PN stored at room temperature for 6-month-aged and freshly prepared electrolytes. Source data are provided as a Source Data file.

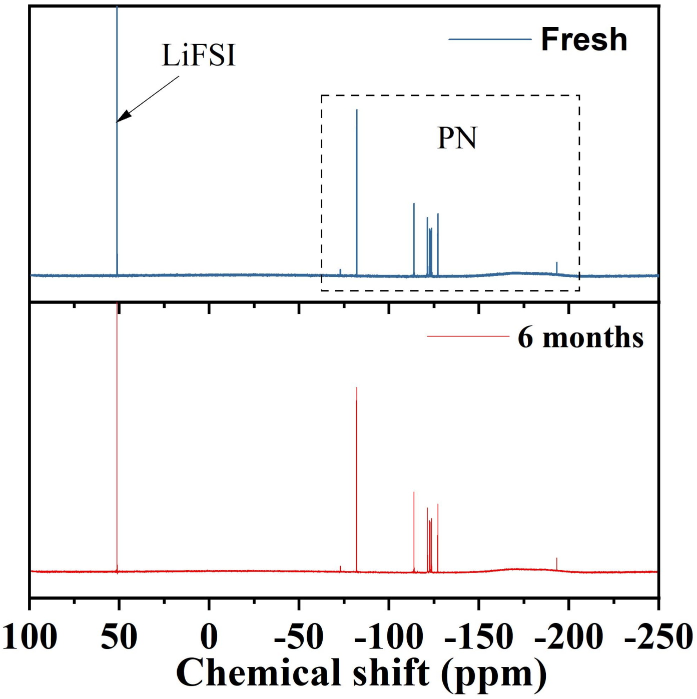

Fig. S4. Comparison of NMR Spectra of electrolytes. ^{19}F NMR spectra of PN stored at room temperature for 6 months and fresh PN. Source data are provided as a Source Data file.

Table S1. Comparison of acidity and moisture content between long-term stored electrolytes and fresh electrolytes

	Fresh	6 months
Free acid content (ppm)	36	38
moisture content (ppm)	12	10

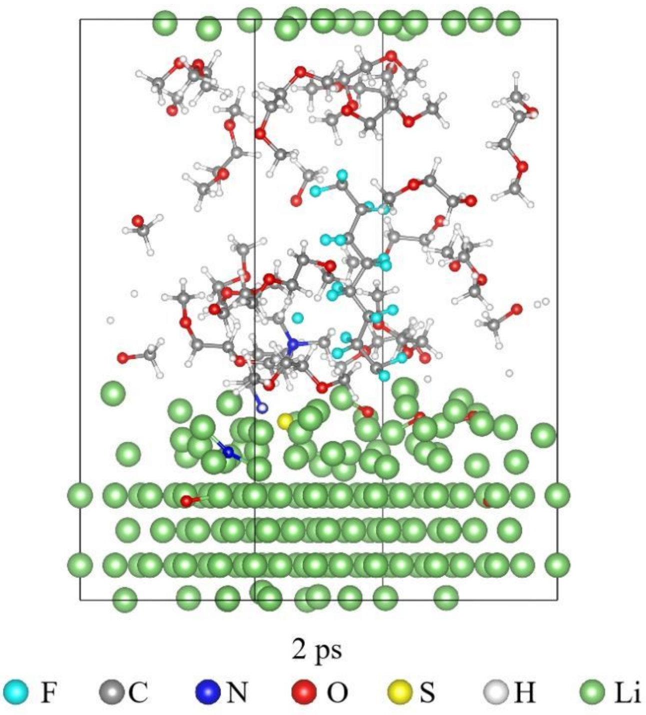

Fig. S5. Simulation of in situ reaction between PN additive and lithium metal. Snapshots from AIMD simulation of decomposition reaction processes between PN with Li metal in a pure DME solvent system at 2 ps. The cyan spheres represent fluorine (F) atoms, the gray spheres represent carbon (C) atoms, the blue spheres represent nitrogen (N) atoms, the red spheres represent oxygen (O) atoms, the yellow spheres represent sulfur (S) atoms, the white spheres represent hydrogen (H) atoms, and the green spheres represent lithium (Li) atoms.

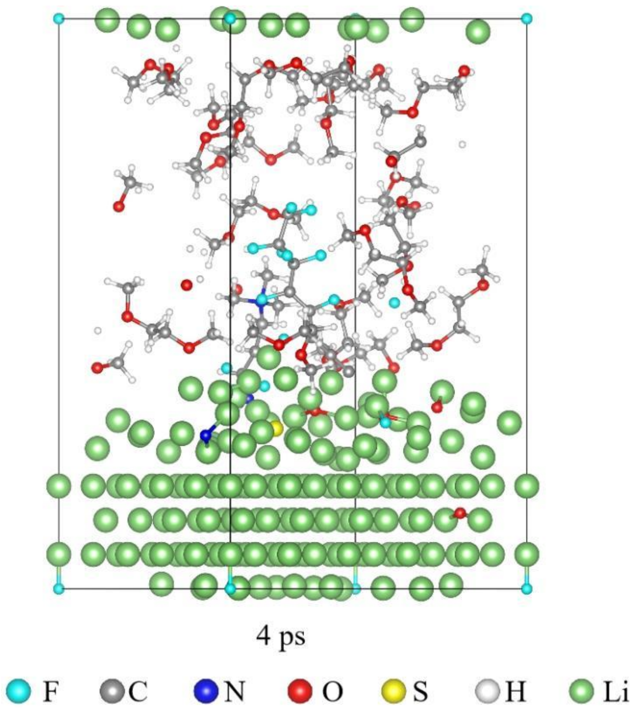

Fig. S6. Simulation of in situ reaction between PN additive and lithium metal. Snapshots from AIMD simulation of decomposition reaction processes between PN with Li metal in a pure DME solvent system at 4 ps. The cyan spheres represent fluorine (F) atoms, the gray spheres represent carbon (C) atoms, the blue spheres represent nitrogen (N) atoms, the red spheres represent oxygen (O) atoms, the yellow spheres represent sulfur (S) atoms, the white spheres represent hydrogen (H) atoms, and the green spheres represent lithium (Li) atoms.

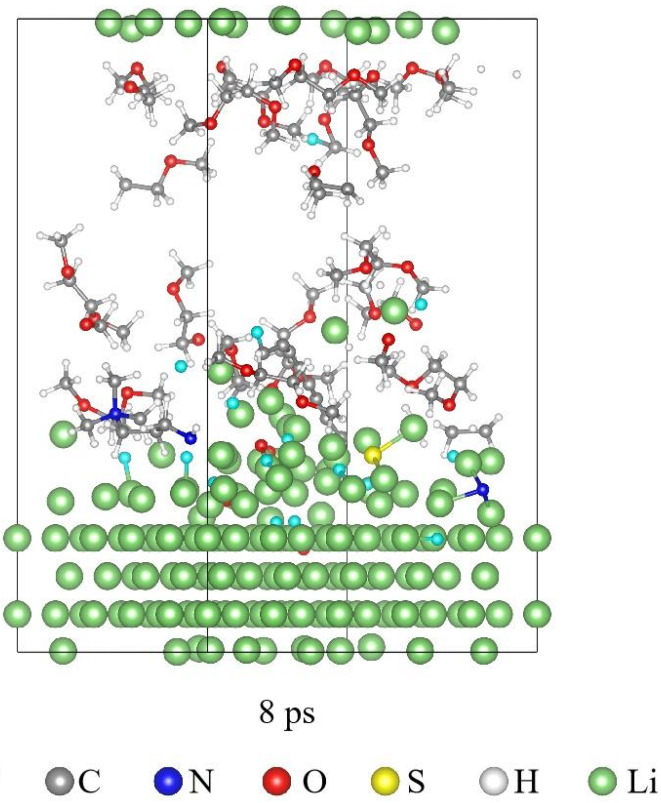

Fig. S7. Simulation of in situ reaction between PN additive and lithium metal. Snapshots from AIMD simulation of decomposition reaction processes between PN with Li metal in a pure DME solvent system at 8 ps. The cyan spheres represent fluorine (F) atoms, the gray spheres represent carbon (C) atoms, the blue spheres represent nitrogen (N) atoms, the red spheres represent oxygen (O) atoms, the yellow spheres represent sulfur (S) atoms, the white spheres represent hydrogen (H) atoms, and the green spheres represent lithium (Li) atoms.

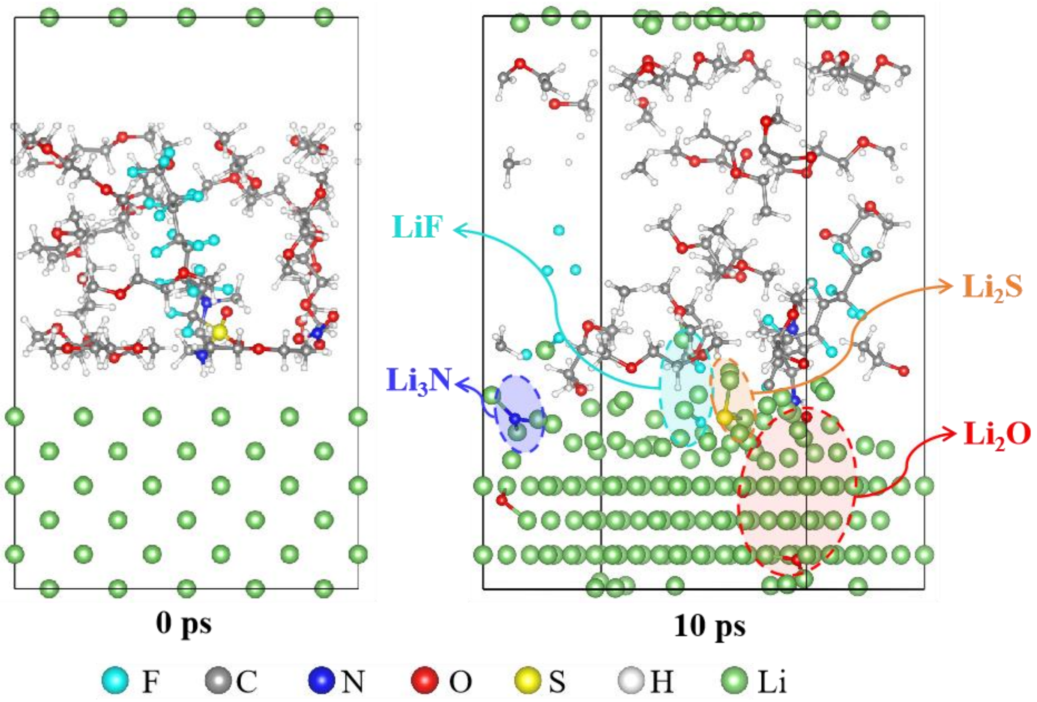

Fig. S8. Simulation of in situ reaction between PN additive and lithium metal. Snapshots from AIMD simulation of decomposition reaction processes between PN with Li metal in a mixed ether solvent system (DEE: DME=9:1 vol%). The cyan spheres represent fluorine (F) atoms, the gray spheres represent carbon (C) atoms, the blue spheres represent nitrogen (N) atoms, the red spheres represent oxygen (O) atoms, the yellow spheres represent sulfur (S) atoms, the white spheres represent hydrogen (H) atoms, and the green spheres represent lithium (Li) atoms. Initial structure of aimd simulation are provided as supplementary data 2.

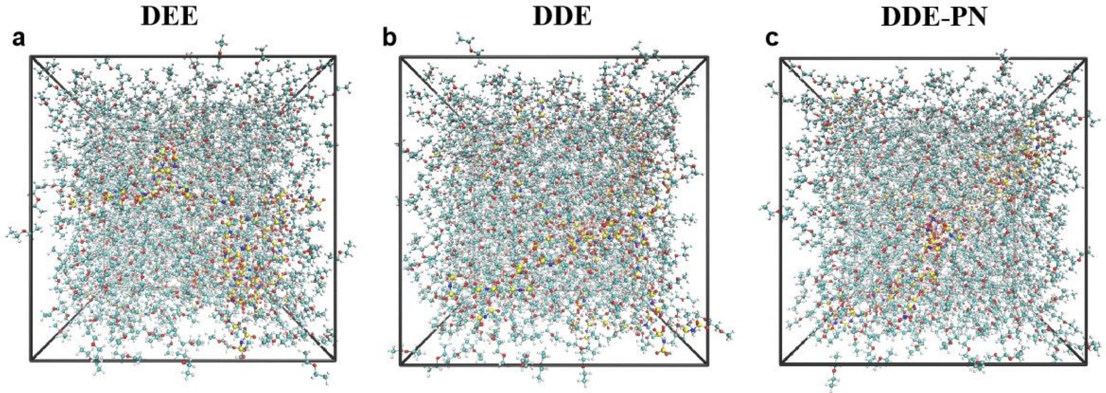

Fig. S9. Simulation of electrolyte solvation structure. The MD snap shot of (a) DEE, (b) DDE and (c) DDE-PN.

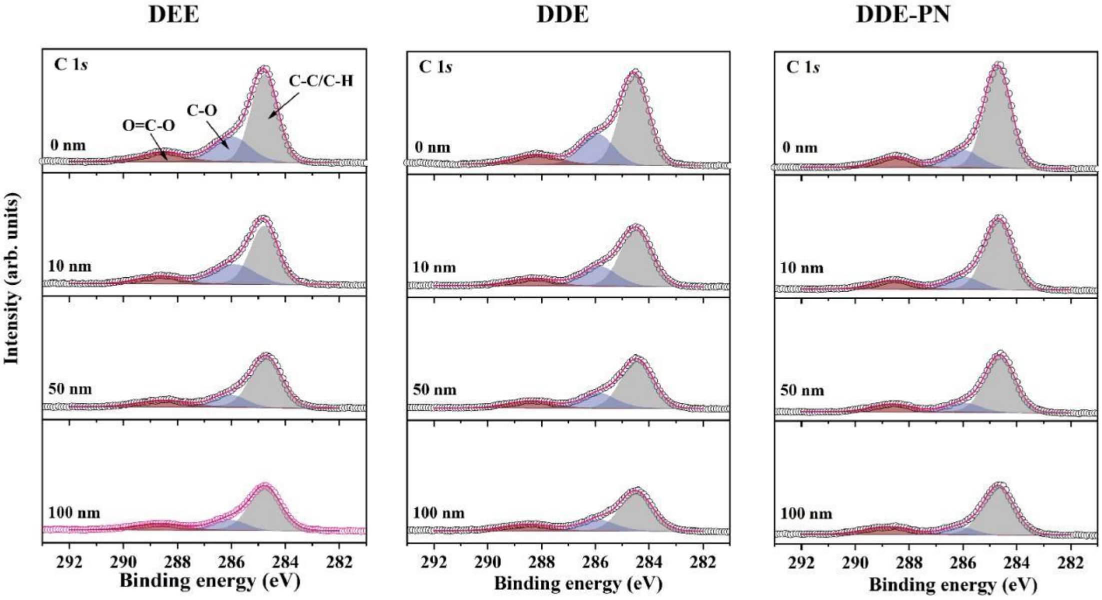

Fig. S10. XPS characterization of SEI chemical composition. XPS depth profiles of C 1*s* of SEI formed on the surface of Cu were measured after depositing lithium metal with a fixed capacity of 2 mAh cm^{-2} at a current density of 1 mA cm^{-2} on Cu in Li||Cu coin cells containing DEE, DDE, and DDE-PN electrolytes after 20 cycles. Source data are provided as a Source Data file.

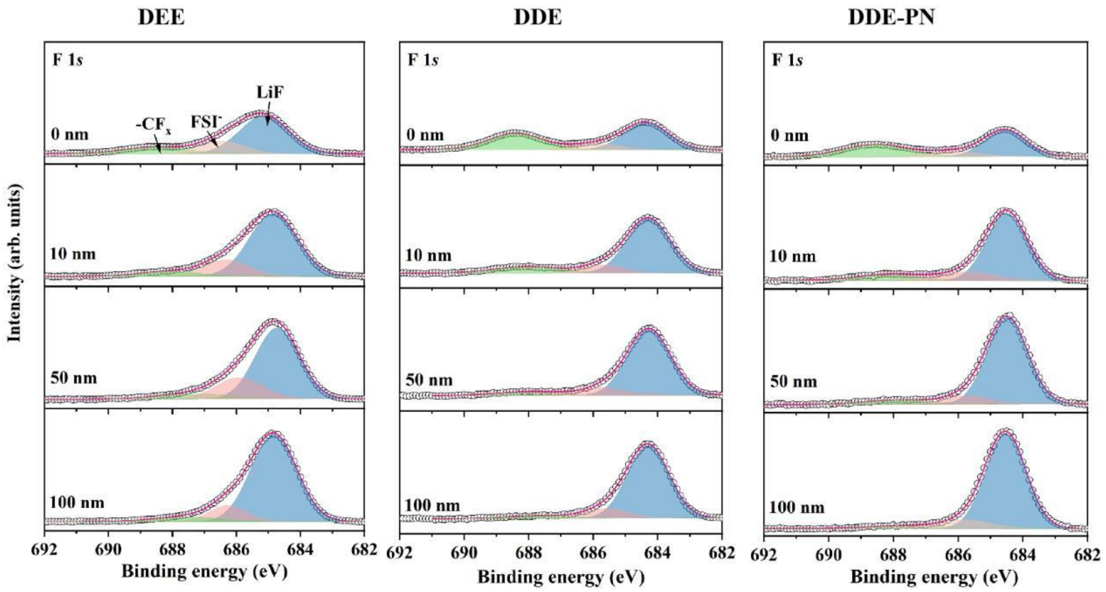

Fig. S11. XPS characterization of SEI chemical composition. XPS depth profiles of F 1*s* of SEI formed on the surface of Cu were measured after depositing lithium metal with a fixed capacity of 2 mAh cm^{-2} at a current density of 1 mA cm^{-2} on Cu in Li||Cu coin cells containing DEE, DDE, and DDE-PN electrolytes after 20 cycles. Source data are provided as a Source Data file.

Source data are provided as a Source Data file.

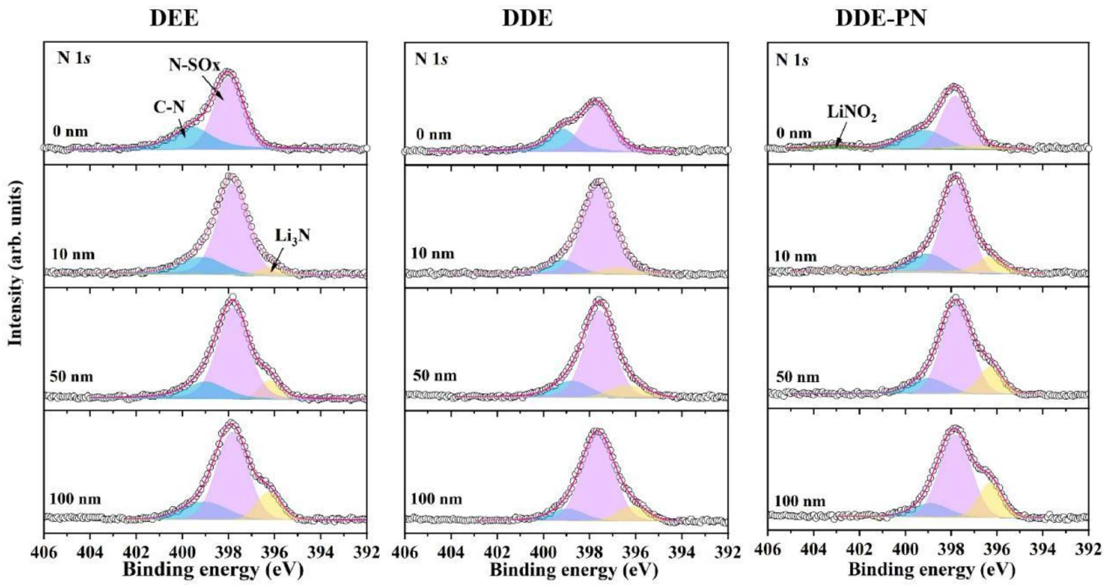

Fig. S12. XPS characterization of SEI chemical composition. XPS depth profiles of N 1*s* of SEI formed on the surface of Cu were measured after depositing lithium metal with a fixed capacity of 2 mAh cm^{-2} at a current density of 1 mA cm^{-2} on Cu in Li||Cu coin cells containing DEE, DDE, and DDE-PN electrolytes after 20 cycles. Source data are provided as a Source Data file. Source data are provided as a Source Data file.

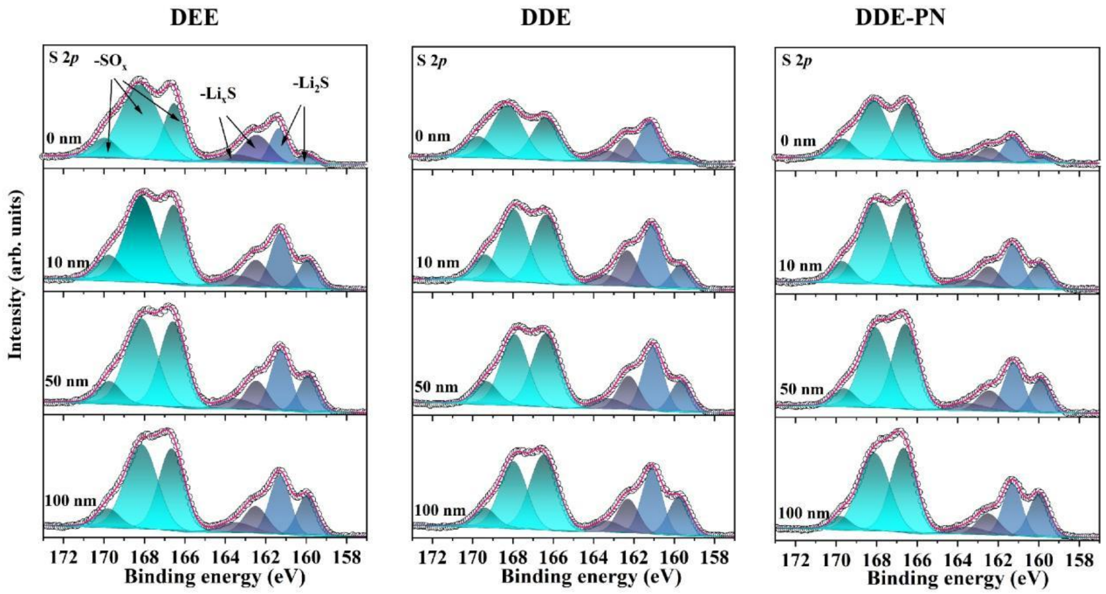

Fig. S13. XPS characterization of SEI chemical composition. XPS depth profiles of S 2*p* of SEI formed on the surface of Cu were measured after depositing lithium metal with a fixed capacity of 2 mAh cm^{-2} at a current density of 1 mA cm^{-2} on Cu in Li||Cu coin cells containing DEE, DDE, and DDE-PN electrolytes after 20 cycles. Source data are provided as a Source Data file. Source data are provided as a Source Data file.

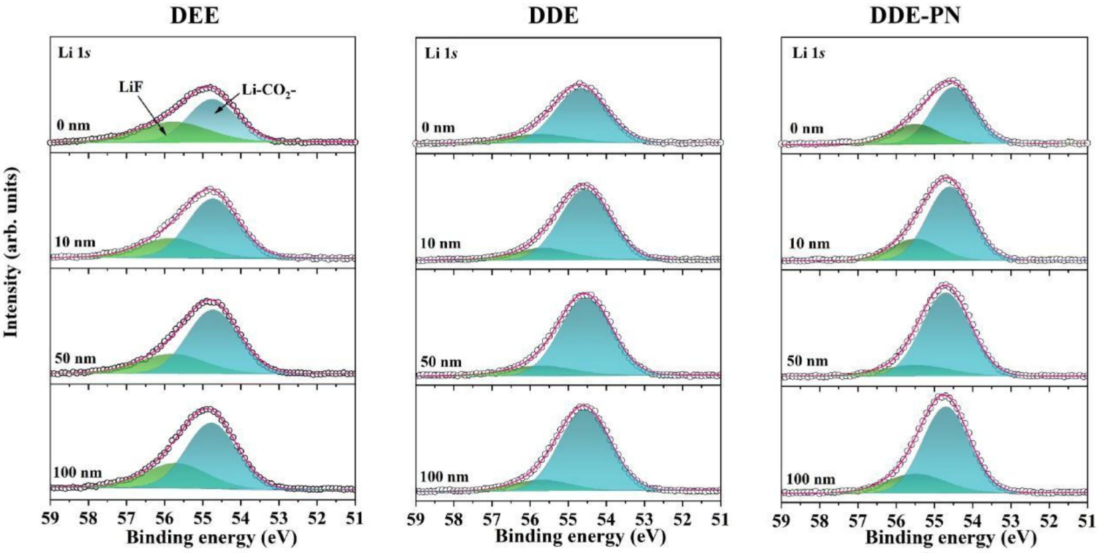

Fig. S14. XPS characterization of SEI chemical composition. XPS depth profiles of Li 1*s* of SEI formed on the surface of Cu were measured after depositing lithium metal with a fixed capacity of 2 mAh cm^{-2} at a current density of 1 mA cm^{-2} on Cu in Li||Cu coin cells containing DEE, DDE, and DDE-PN electrolytes after 20 cycles. Source data are provided as a Source Data file. Source data are provided as a Source Data file.

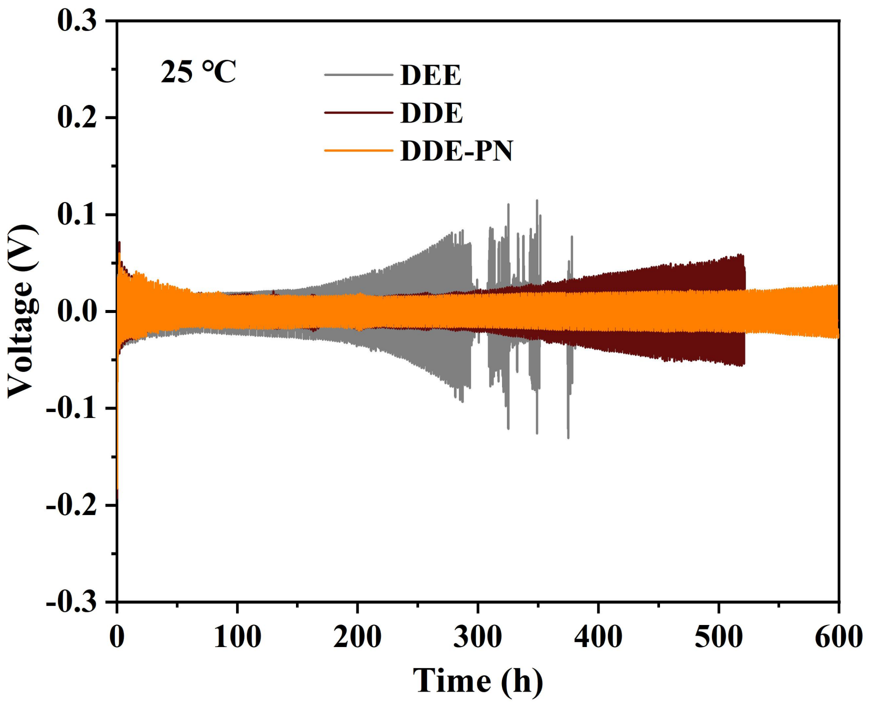

Fig. S15. Li plating/stripping performance of lithium metal anode. Li plating/stripping performance of Li||Li cells under 25 °C. The current density is 1 mA cm^{-2} and the plating capacity is 1 mAh cm^{-2}. Source data are provided as a Source Data file.

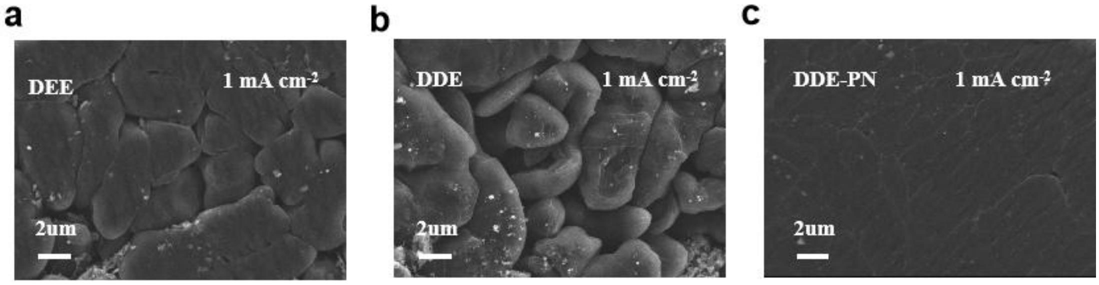

Fig. S16. Morphology of lithium metal deposition. Li plating morphology on copper working electrodes with a capacity of 2 mAh cm^{-2} at 1 mA cm^{-2} after 5 cyclesin the (a) DEE, (b) DDE and (c) DDE-PN electrolyte under 25 ℃.

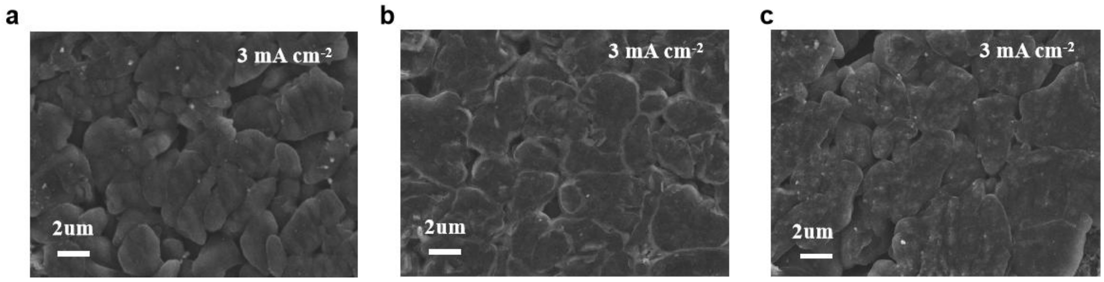

Fig. S17. Morphology of lithium metal deposition. Li plating morphology on copper working electrodes with a capacity of 2 mAh cm^{-2} at 3 mA cm^{-2} after 5 cycles in the (a) DEE, (b) DDE and (c) DDE-PN electrolyte under 25 ℃.

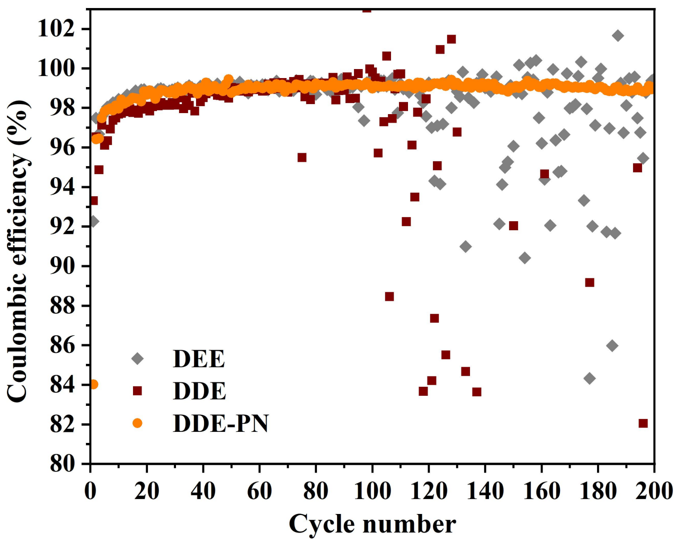

Fig. S18. Electrochemical performance of lithium metal anode. Cycling CE of Li||Cu cells. The current density is 1 mA cm^{-2} and the plating capacity is 1 mAh cm^{-2} under 25 °C. Source data are provided as a Source Data file.

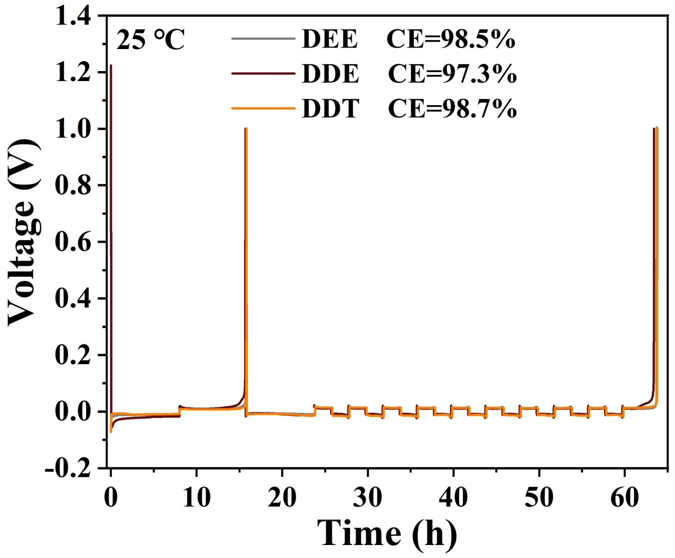

Fig. S19. Electrochemical performance of lithium metal anode. Aurbach CE test of Li||Cu cells under 25 °C. Source data are provided as a Source Data file.

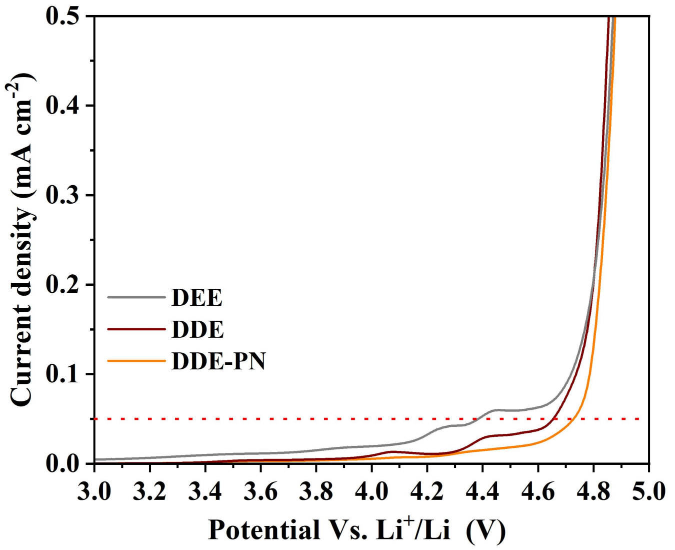

Fig. S20. Evaluation of electrolyte oxidation stability. Oxidative stability measured via LSV for Li||carbon-coated Al cells. Scan rate: 5mV s^{-1}, 25 °C. Source data are provided as a Source Data file.

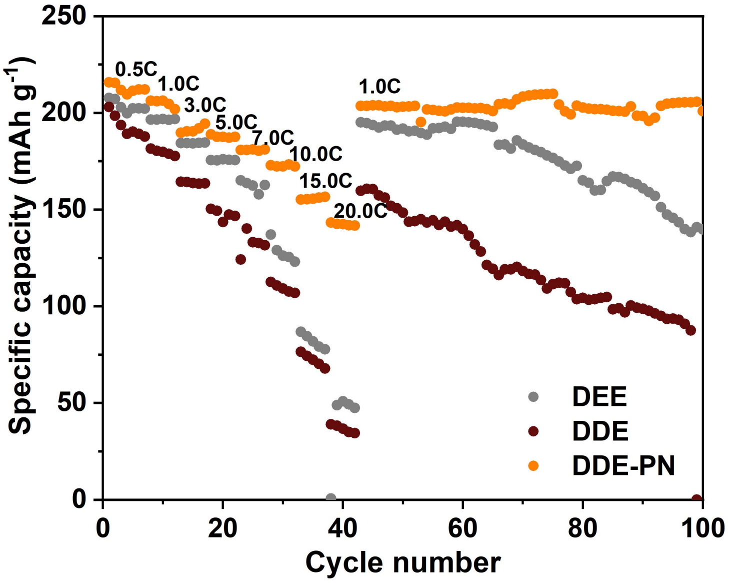

Fig. S21. Electrochemical performance evaluation of cathode. Rate capability of Li||NMC811 cells under different charging/discharging rates. 1.0 C = 200 mA g^{-1} . Source data are provided as a Source Data file.

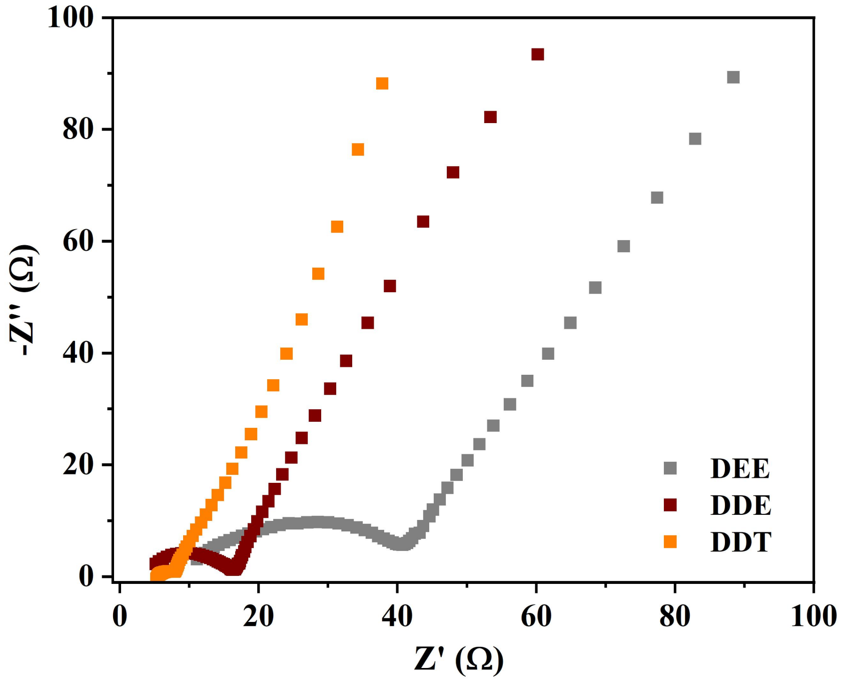

Fig. S22. Evaluation of Interface Stability during cycling. Impedance spectra of the NMC811 electrode after 5 cycles at 1.0 C under 25 ℃ using different electrolytes. Source data are provided as a Source Data file.

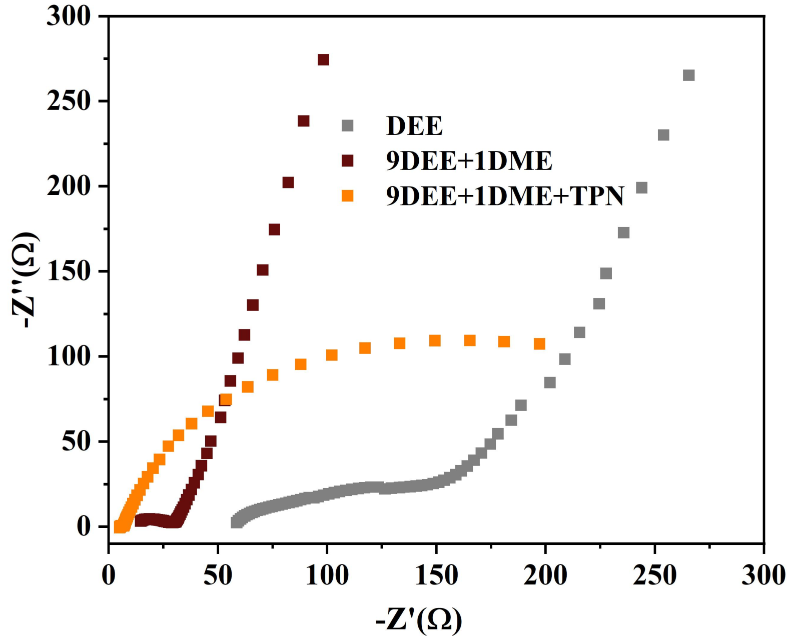

Fig. S23. Evaluation of Interface Stability during cycling. Impedance spectra of the NMC811 electrode after 100 cycles at 1.0 C under 25 ℃ using different electrolytes. Source data are provided as a Source Data file.

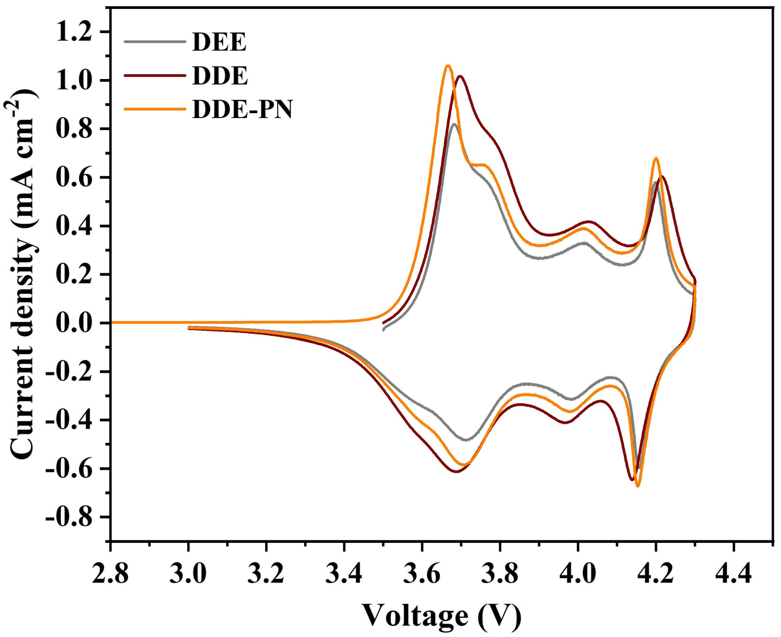

Fig. S24. Evaluation of cycling stability of different electrolytes. Cyclic voltammetry curves of the NMC811 cathode after 5 cycles using different electrolytes with a scan rate of 0.1 mV s^{-1}, voltage range of 3-4.3V, and temperature of 25 °C. Source data are provided as a Source Data file.

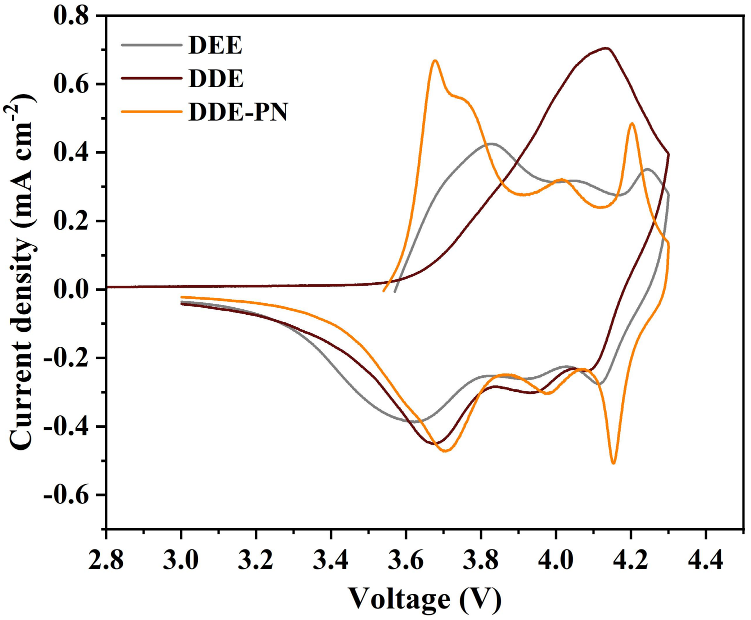

Fig. S25. Evaluation of cycling stability of different electrolytes. Cyclic voltammetry curves of the NMC811 cathode after 100 cycles using different electrolytes with a scan rate of 0.1 mV s^{-1}, voltage range of 3.0-4.3V, and temperature of 25 °C. Source data are provided as a Source Data file.

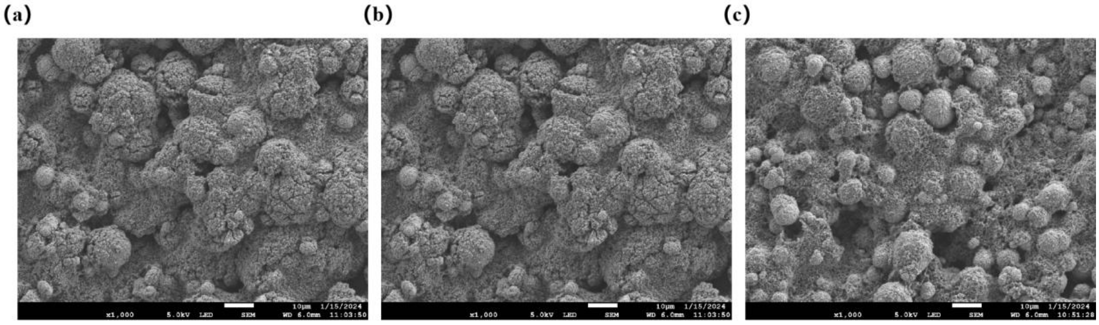

Fig. S26. Morphology of NMC811 particles after long cycling. SEM images of the NCM811 cathode morphology from the cell after 100 cycles at 1.0 C, a voltage range of 3.0–4.3 V in the (a) DEE, (b) DDE and (c) DDE-PN electrolyte under 25 ℃.

.

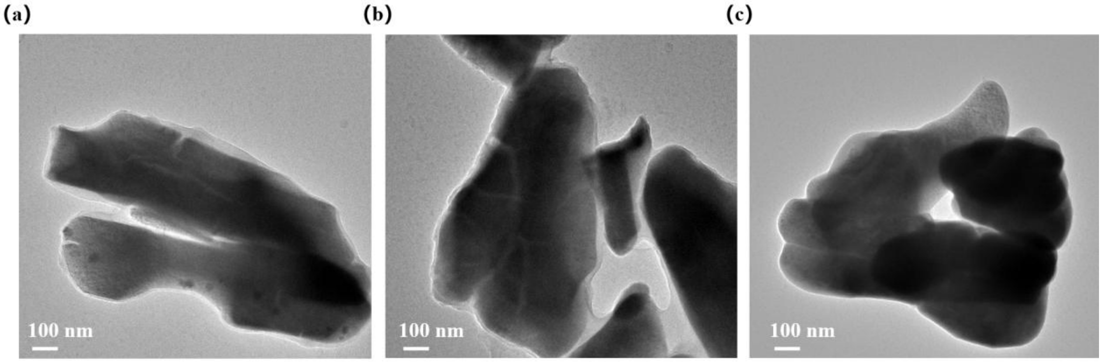

Fig. S27. Morphology of NMC811 particles after long cycling. TEM images of the NCM811 cathode morphology from the cell after 100 cycles at 1.0 C, a voltage range of 3.0–4.3 V in the (a) DEE, (b) DDE and (c) DDE-PN electrolyte under 25 ℃.

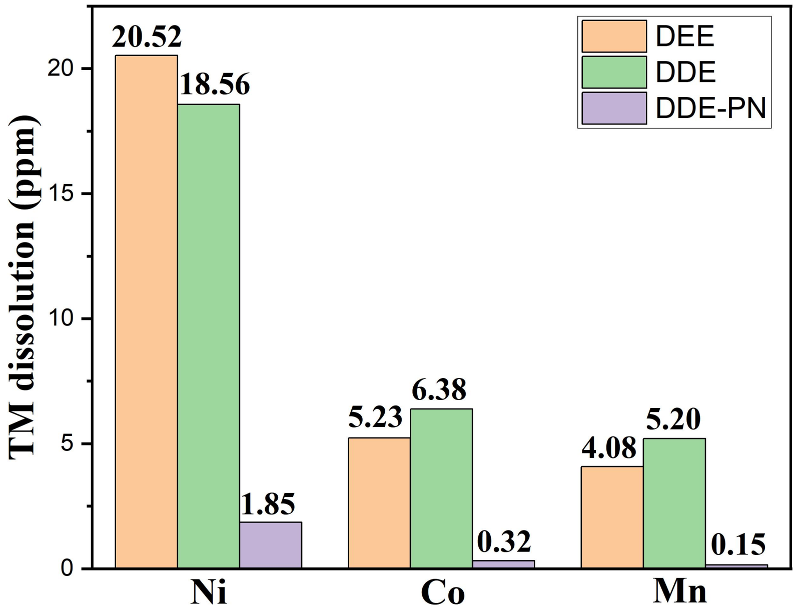

Fig. S28. Characterization of cathode Stability. Transition metal (TM) dissolution measured by inductively coupled plasma mass spectrometry (ICP-MS) after 50 cycles at 1.0 C, a voltage range of 3.0–4.3 V in the DEE, DDE and DDE-PN electrolyte under 25 °C.

.

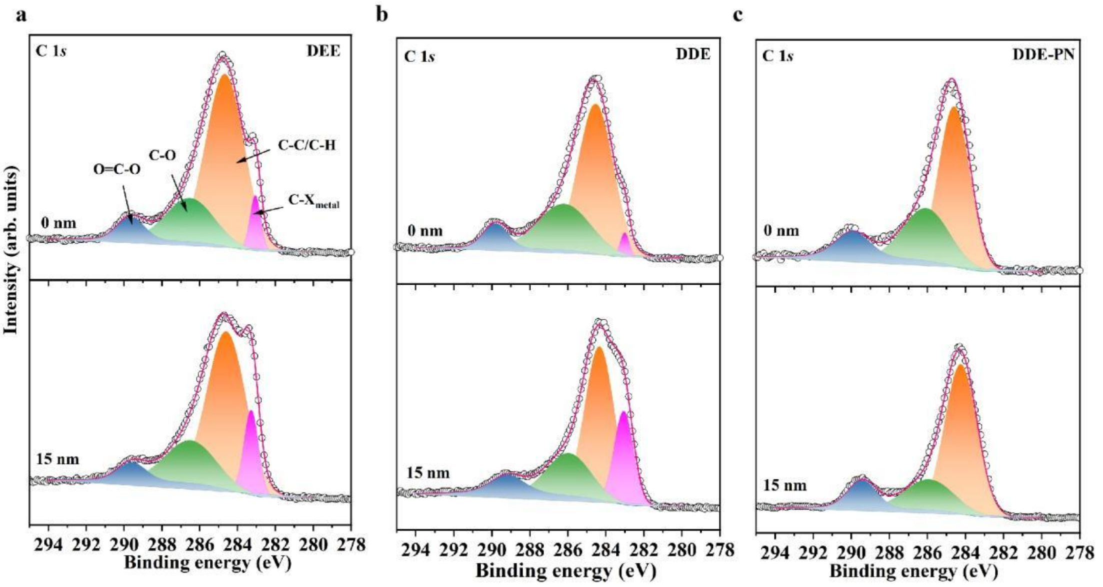

Fig. S29. Characterization of CEI compositions. XPS depth profiles of C 1*s* of CEI formed on NMC811 electrode surface after 50 cycles at 1.0 C, a voltage range of 3.0–4.3 V in Li||NMC811 coin cells with (a) DEE, (b) DDE and (c) DDE-PN electrolyte under 25 ℃. Source data are provided as a Source Data file.

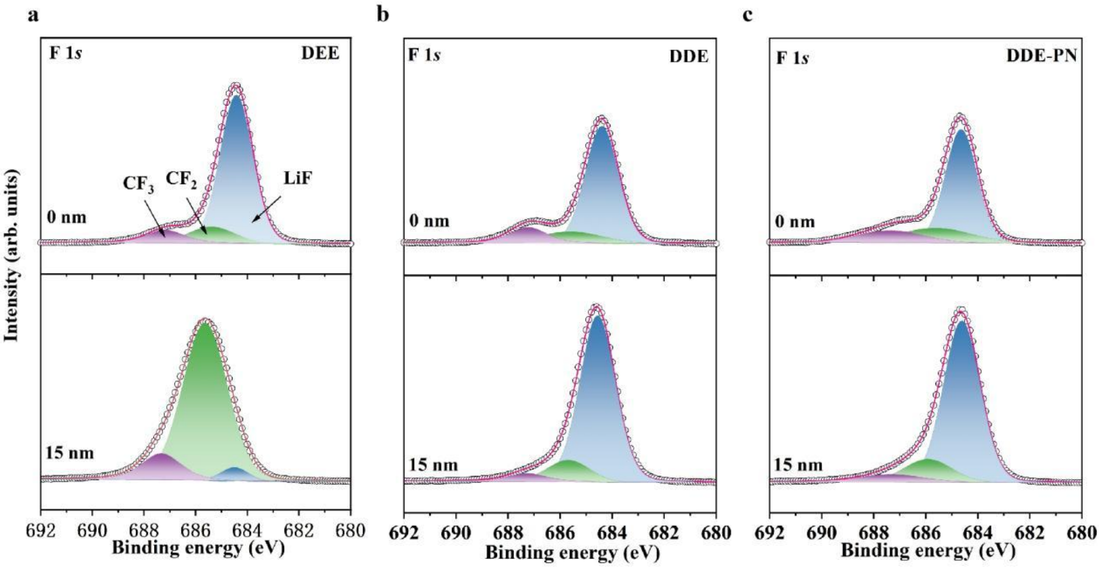

Fig. S30. Characterization of CEI compositions. XPS depth profiles of F 1*s* of CEI formed on NMC811 cathode surface after 50 cycles at 1.0 C, a voltage range of 3.0–4.3 V in Li||NMC811 coin cells with (a) DEE, (b) DDE and (c) DDE-PN electrolyte under 25 ℃. Source data are provided as a Source Data file.

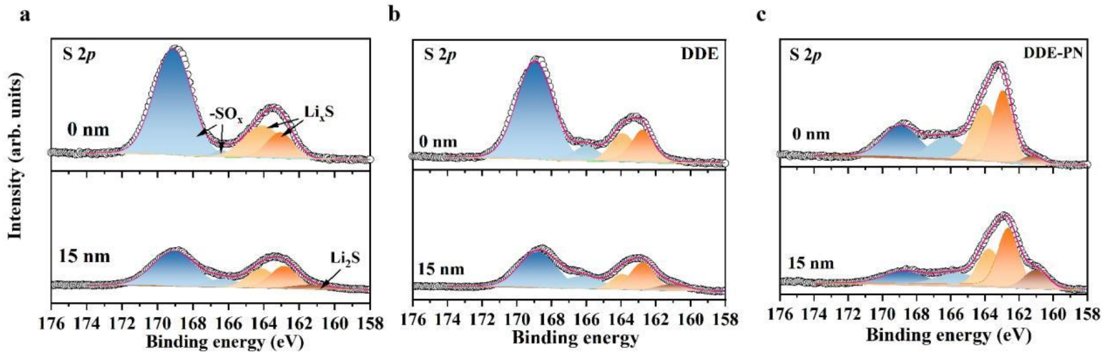

Fig. S31. Characterization of CEI compositions. XPS depth profiles of S 2*p* of CEI formed on NMC811 surface after 50 cycles at 1.0 C, a voltage range of 3.0–4.3 V in Li||NMC811 coin cells with (a) DEE, (b) DDE and (c) DDE-PN electrolytes under 25 ℃. Source data are provided as a Source Data file.

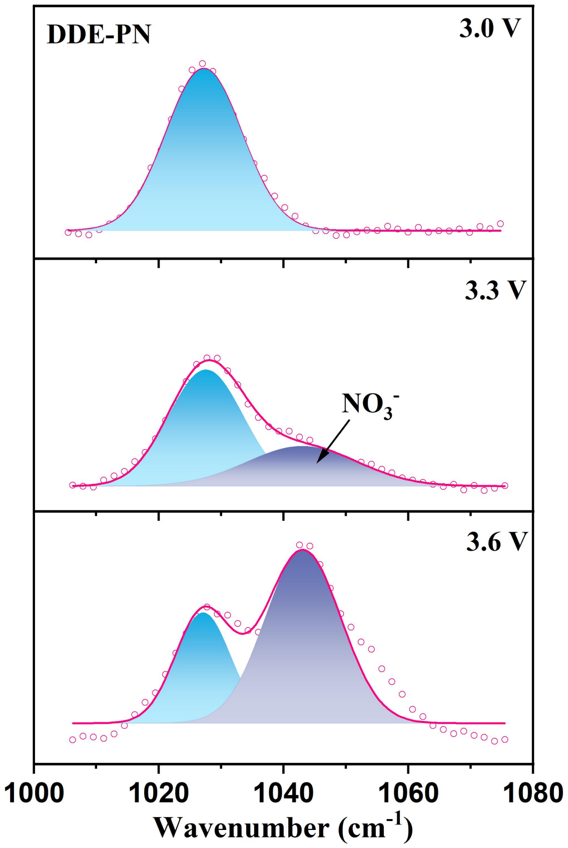

Fig. S32. ATR-SEIRAS of the surface layer of the working electrode under different voltages. Raman spectra of NO_3^- on the working electrode under different voltages in DDE-PN electrolytes. Source data are provided as a Source Data file.

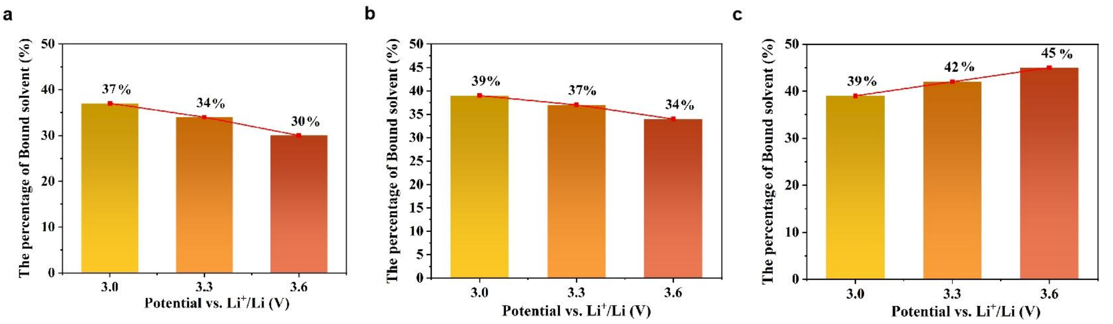

Fig. S33. ATR-SEIRAS of the surface layer of the working electrode under different voltages. The variation trend of Bound-solvent in the double layer with respect to voltage in (a) DEE, (b) DDE and (c) DDE-PN electrolytes .

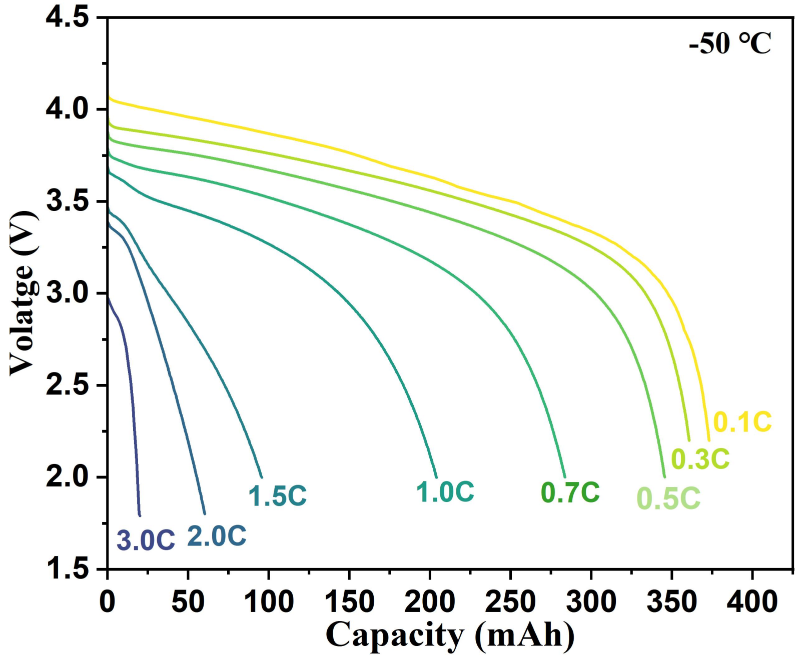

Fig. S34. **Electrochemical performance of pouch cell at low temperatures.** Typical voltage profiles of the Li||NMC811 pounch cells with various currents ranging from 0.1 C (0.4 mA cm^{-2}) to 3.0 C (12 mA cm^{-2}) under -50 °C. Source data are provided as a Source Data file.

Table S2. Comparison of our work with recent works on low-temperature lithium-based pouch cells.

Electrolyte	Cell condition	Cutoff Potential （vs Li/Li$^+$）	Cell capacity	(Maximum) Specific energy at low temperature	(Maximum) Specific power at low temperature	Refs.
1M LiFSI in DEE	Li‖SPAN pouch cell	3.5 V	160 mAh	143 Wh kg^{-1} at -40 ℃	17.2 W kg^{-1} at -40 ℃ (calculated)	Ref.1
1 M LiFSI in BTFE/DME (5 : 1 vol%)	Li‖NMC811 pouch cell	4.3 V	5 Ah	230 Wh kg^{-1} at -40 ℃	23 W kg^{-1} at -40 ℃ (calculated)	Ref.2
1.0 M LiFSI in DiFEC/MTFC/HFME (1:2:2 vol%)	Graphite‖NMC523 pouch cell	4.6 V	2 Ah	270 Wh kg^{-1} at -40 ℃	16.5 W kg^{-1} at -40 ℃ (calculated)	Ref.3
0.6 M LiFSI + 0.4 M LiDFOB in DMS	LCO‖Graphite pouch cell	4.3 V	1 Ah	160 Wh kg^{-1} at -50 ℃	19.3 W kg^{-1} at -50 ℃ (calculated)	Ref.4

$LiPF_6$ in PC/TFEP/ EMC	NMC811‖Graphite pouch cell	4.3 V	1 Ah	221 Wh kg^{-1} at -40 ℃ (calculated)	22.1 W kg^{-1} at -40 ℃ (calculated)	Ref.5
DDE-PN	NMC811‖Li pouch cell	4.3 V	0.5 Ah	308.9 Wh kg^{-1} at -50 ℃	938.5 W kg^{-1} at -50 ℃	This work

Table S3. Weight of the 500 mAh Li||NMC811 pouch cells components for calculating the specific energy and specific power.

Components	Weights (g)
Electrode materials and separator	3.05
electrolyte	1.25
Packing foil and tabs	1.81
Total weight	6.11

Supplementary references:

[1] J. Holoubek, H. Liu, Z. Wu, Y. Yin, X. Xing, G. Cai, S. Yu, H. Zhou, T. A. Pascal, Z. Chen, P. Liu, Tailoring electrolyte solvation for Li metal batteries cycled at ultra-low temperature. Nat. Energy 6, 303-313 (2021).

[2] J. Holoubek, K. Kim, Y. Yin, Z. Wu, H. Liu, M. Li, A. Chen, H. Gao, G. Cai, T. A. Pascal, P. Liu, Z. Chen, Electrolyte design implications of ion-pairing in low-temperature Li metal batteries. Energy Environ. Sci. 15, 1647-1658 (2022).

[3] X. Zheng, Z. Cao, W. Luo, S. Weng, X. Zhang, D. Wang, Z. Zhu, H. Du, X. Wang, L. Qie, H. Zheng, Y. Huang, Solvation and Interfacial Engineering Enable −40 °C Operation of Graphite/NCM Batteries at Energy Density over 270 Wh kg^{-1}. Adv. Mater. 35, 2210115 (2023).

[4] Y. Zhao, Z. Hu, Z. Zhao, X. Chen, S. Zhang, J. Gao, J. Luo, Strong Solvent and Dual Lithium Salts Enable Fast-Charging Lithium-Ion Batteries Operating from −78 to 60 °C. J. Am. Chem. Soc. 2023, 145, 22184-22193.

[5] L. Chen, J. Wang, M. Chen, Z. Pan, Y. Ding, Z. Song, X. Ai, Y. Cao, Z. Chen, “Dragging effect” induced fast desolvation kinetics and -50 ℃ workable high-safe lithium batteries. Energy Storage Mater. 65, 103098 (2024).
