# Supplementary material for: Multifunctional electrolyte additive for high power lithium metal batteries at ultra-low temperatures
Source: Nat Commun. 2025 Apr 8;16:3344. doi: 10.1038/s41467-025-58627-3 (PMC11978743; doi:10.1038/s41467-025-58627-3)
Supplement: Supplementary file 3 — Description of Additional Supplementary Files [file 41467_2025_58627_MOESM3_ESM.pdf]

<u>Description of Additional Supplementary Files</u>

Supplementary Data 1：Initial structure of aimd simulation in pure DME solvent system

Supplementary Data 2：Initial structure of aimd simulation in a mixed ether solvent system (DEE: DME=9:1 vol%)
